# Public health surveillance in the U.S. Department of Veterans Affairs: evaluation of the Praedico surveillance system

**DOI:** 10.1186/s12889-022-12578-2

**Published:** 2022-02-10

**Authors:** Cynthia Lucero-Obusan, Gina Oda, Anoshiravan Mostaghimi, Patricia Schirmer, Mark Holodniy

**Affiliations:** 1 U.S. Department of Veterans Affairs, Veterans Health Administration, Patient Care Services, Public Health Program Office, Washington, DC, Palo Alto, CA USA; 2 Division of Infectious Diseases & Geographic Medicine, Stanford University, Stanford, CA USA

**Keywords:** Big data, Health informatics, Public health, Surveillance, Syndromic surveillance, System evaluation, Electronic health records, Veterans

## Abstract

**Background:**

Early threat detection and situational awareness are vital to achieving a comprehensive and accurate view of health-related events for federal, state, and local health agencies. Key to this are public health and syndromic surveillance systems that can analyze large data sets to discover patterns, trends, and correlations of public health significance. In 2020, Department of Veterans Affairs (VA) evaluated its public health surveillance system and identified areas for improvement.

**Methods:**

Using the Centers for Disease Control and Prevention (CDC) Guidelines for Evaluating Public Health Surveillance Systems, we assessed the ability of the Praedico Surveillance System to perform public health surveillance for a variety of health issues and evaluated its performance compared to an enterprise data solution (VA Corporate Data Warehouse), legacy surveillance system (VA ESSENCE) and a national, collaborative syndromic surveillance platform (CDC NSSP BioSense).

**Results:**

Review of system attributes found that the system was simple, flexible, and stable. Representativeness, timeliness, sensitivity, and Predictive Value Positive were acceptable but could be further improved. Data quality issues and acceptability present challenges that potentially affect the overall usefulness of the system.

**Conclusions:**

Praedico is a customizable surveillance and data analytics platform built on big data technologies. Functionality is straightforward, with rapid query generation and runtimes. Data can be graphed, mapped, analyzed, and shared with key decision makers and stakeholders. Evaluation findings suggest that future development and system enhancements should focus on addressing Praedico data quality issues and improving user acceptability. Because Praedico is designed to handle big data queries and work with data from a variety of sources, it could be enlisted as a tool for interdepartmental and interagency collaboration and public health data sharing. We suggest that future system evaluations include measurements of value and effectiveness along with additional organizations and functional assessments.

## Background

Public health surveillance involves the ongoing collection, analysis, and interpretation of health-related data. These data are used to plan and implement health policy, evaluate public health practice, track diseases, disseminate information and monitor naturally occurring or intentional biological and environmental threats [1–3]. Considerable time and resources have been spent by federal agencies, including the Centers for Disease Control and Prevention (CDC), as well as state and local health jurisdictions to develop public health surveillance systems in use today [3].

The Veterans Health Administration (VHA) is the largest integrated healthcare system in the U.S., providing care to over 9 million enrolled Veterans at 171 medical centers and 1283 outpatient sites across all U.S. states, territories, the District of Columbia, and the Philippines [4]. VHA was a pioneer as one of the first major healthcare systems to adopt an electronic health record (EHR), known as the Veterans Information Systems and Technology Architecture (VistA) [5, 6]. Because Veterans may obtain healthcare from different services and different Department of Veterans Affairs (VA) medical centers, an integrated and complete view of their health-related data is needed.

In 2006, VA developed the Corporate Data Warehouse (CDW), a Structured Query Language (SQL) relational database (RDB) with national data [7, 8]. CDW is a repository and health data warehouse comprised of VistA clinical data (via VistA journal files) and other data systems (enrollment, administrative, financial, utilization, benefits and more). Data in CDW is extracted from source VistA EHR and minimally transformed to preserve relational data model constraints. Aggregate data reports can be visualized with the Pyramid 2019 (Pyramid Analytics) user interface or Microsoft Power Business Intelligence (BI) software [9, 10]. Patient-level data is queried using Microsoft’s SQL Server Management Studio and can also be viewed and analyzed via Pyramid Analytics, Power BI, or other programs [9–11].

VHA’s first electronic public health surveillance system was the Electronic Surveillance System for the Early Notification of Community-based Epidemics (ESSENCE), adopted in 2004-2005 and developed by Johns Hopkins University [12, 13]. ESSENCE utilizes a point-and-click user interface allowing queries to be made against its data feed. It also has data analysis, alerting and visualization software, including geospatial mapping. A version of ESSENCE (VA ESSENCE) was adapted for VHA data to receive data daily from mainframe servers via Health Level Seven International (HL7) formatted flat files for a limited selection of VistA data domains and remained in use for 10 years.

Praedico (Bitscopic Inc.) was launched in VHA in 2015. Praedico uses the Bitscopic Data Platform (BDaP), a data extraction-transformation framework capable of extracting heterogenous data from diverse legacy and modern electronic health data sources into a big data accessible central data lake [14]. Key improvements included having an enterprise solution utilizing big data architecture; VistA data extraction from a wider complement of data domains; as well as processing, storage, exploration, analytics and visualization features in one integrated platform.

### Public Health Importance & Purpose

A national public health surveillance strategy calls for cooperation on a federal, state, and local level in combination with private sector entities and non-governmental organizations [15]. The purpose of public health surveillance systems is to analyze large data sets to discover patterns or trends which ultimately serve to facilitate the prevention or control of diseases. Key to this are systems deemed to be flexible, easy to use, secure, reliable and cost-effective to give decision makers timely access to the data they need. Surveillance is an important activity for VHA and encompasses infectious diseases, chronic illnesses, natural disasters, environmental exposures, plus biologic and chemical threats. It also includes syndromic surveillance for early detection of outbreaks and monitoring of disease trends. Surveillance data meets the definition of big data, due to the large volume of data to be evaluated, the wide variety of structured and unstructured data sources, and the rate at which data are generated and needed for analysis [16–18]. Praedico is a surveillance system used by VHA Public Health Program Office (PHPO) epidemiologists, designed to collect, and analyze public health data across multiple EHR domains with a focus on situational awareness and early detection. Interpretation of health data by expert users of the Praedico system allows VHA PHPO to monitor diseases, including outbreaks and events of public health significance, and disseminate information to providers and VA leadership. Principle objectives of the Praedico system include surveillance for known diseases of interest (both infectious and non-infectious), syndromic surveillance, emerging infections and to facilitate epidemiologic investigations conducted by PHPO.

System evaluation is a critical task to ensure appropriate monitoring of important conditions and diseases. The goal of this analysis was to assess the ability of Praedico to perform public health surveillance and evaluate its performance compared to an enterprise data solution (CDW), legacy surveillance system (VA ESSENCE) and a national, collaborative surveillance platform [CDC National Syndromic Surveillance Program (NSSP) BioSense Platform]. This evaluation may also be beneficial to other agencies and jurisdictions in evaluating their public health and syndromic surveillance systems.

## Methods & analysis

The operation of Praedico was assessed by VHA epidemiologists who are subject matter experts (SMEs) in infectious diseases, surveillance, and public health informatics, using criteria from CDC’s 2001 Updated Guidelines for Evaluating Public Health Surveillance Systems [19]. This evaluation framework has been in use for 20  years and evaluated against existing approaches [20–24]. The evaluation begins with a description of the system, its operation, and data sources. Next, usefulness and nine surveillance attributes of the system are assessed. All attributes were evaluated qualitatively, and five attributes were evaluated, at least in part, quantitatively [timeliness, stability, data quality, sensitivity, and predictive value positive (PVP)]. To evaluate sensitivity and PVP we used data from VA ESSENCE, CDW and/or raw VistA extracts as “gold standards”, using methods previously described [25]. Data quality and representativeness assessments also utilized CDW data for comparison. Additional comparisons (timeliness and representativeness) were conducted between Praedico and the CDC BioSense platform (NSSP ESSENCE system) [26]. In the fall of 2020 (following a 3-year hiatus), VA resumed transmission of a limited data set of emergency department and urgent care data to the CDC BioSense platform for syndromic and electronic disease surveillance.

Permission to access data was granted through the VHA Director of National Data Systems, Austin Information Technology Center, in accordance with the Privacy Act of 1974; System of Records entitled “National Patient Databases-VA” (121VA10A7). The data utilized for this evaluation were obtained only for the purpose of public health operations in VHA [27].

### System Description & Operation

Praedico is a customizable surveillance, analytics, reporting, visualization, and data management platform built on big data technologies. Praedico is used by PHPO for syndromic surveillance (including influenza-like-illness [28, 29] and other syndromes to monitor weekly trends and alerts for potential outbreaks), routine PHPO surveillance activities (including influenza and RSV surveillance [29–34] and biweekly influenza reports for VHA leadership and the field), and additional ad hoc surveillance, special studies, or other public health operational activities, including monitoring for emerging or re-emerging infectious diseases [35–37], comparative effectiveness of influenza vaccines [38], a SARS-CoV-2 prediction model [39], an evaluation of COVID-19 testing practices [40], and mycobacterial reviews and epidemiological investigations [41–44].

There are three components to Praedico: integration, discovery, and analytics. The integration layer contains a data source connector that can be used to access data from disparate sources such as VistA, telephone triage and CDW as well as non-VHA data sources. Extracted data are integrated to populate the discovery layer. Here data is processed, which includes scrubbing, correlating, standardizing, normalizing, conflating, and deduplication using a combination of pre-defined business intelligence rules and machine learning algorithms. Finally, the analytics layer is composed of a user interface that allows users to query against the database.

### Data sources & resources

Praedico consumes data and allows for the secure ingestion, integration, manipulation, analysis, visualization and sharing of sizable and diverse data sets. It can be used in combination with or in lieu of a traditional RDB. Praedico is integrated into a commercially available big data platform (Cloudera), enabling it to integrate with other big data technologies (e.g. Hive, HBase, and Spark).

**Fig. 1:**
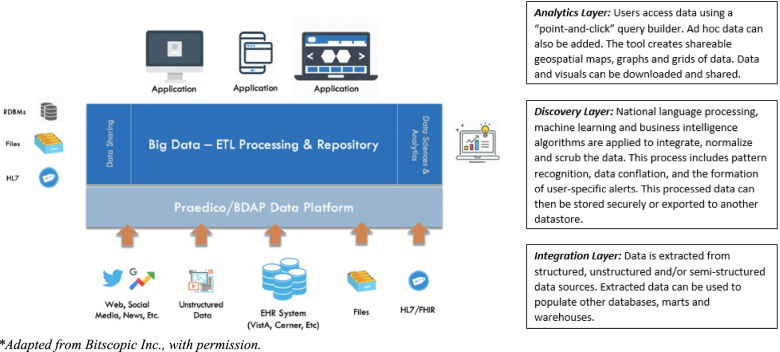
Analytics Engine for Bitscopic Platform*. Praedico is composed of three layers: integration, discovery and analytics

Praedico’s data extraction subsystem pulls data from multiple EHR domains from VistA as well as other VHA sources (such as *DSHI TriageXpert™* telephone triage system database). The data gathering process begins with the BDaP data extractor and transformer, which pulls data into an Oracle database. A data loader called Sqoop™ (Apache Software Foundation) then pulls transformed data into a 9 node Cloudera/Hadoop Distributed File system (HDFS) data repository (VA data lake). Finally, a data bulk loader called Phoenix (Apache Software Foundation) loads the data from the data lake into HBase (Apache Software Foundation), a non-relational, distributed big data store subsystem. Praedico has the ability to connect to other enterprise data warehouses (such as CDW), pull data from unstructured data sources (e.g. social media) and import data from outside sources. Figure 1 illustrates the layers and flow of data through Praedico.

### Usefulness

The principal usefulness of Praedico is that enables PHPO epidemiologists to access, analyze, interpret and ultimately to act upon critical public health data. Data can be analyzed using Praedico’s dashboard or exported to other applications for report generation and timely communication of findings. Praedico has integrated visualization software, including geospatial mapping and graphing capabilities. Extracted and/or processed data can be used to populate other data sources (e.g. data warehouses). International Classification of Diseases, Ninth and Tenth Revisions, Clinical Modification (ICD-9-CM and ICD-10-CM) diagnoses are categorized using predefined syndrome definitions used by PHPO epidemiologists for syndromic surveillance activities. Queries are built using drop-down menus where users select pre-populated terms for inclusion, input wildcard expressions or use an advanced query tool for complex queries. This balances the need for timesaving, straightforward querying while still giving users the ability to write more complex queries. Lastly, Praedico has alerting capabilities that utilize adaptable machine learning algorithms and a proprietary decision tree classifier which prompts users to review and prioritize data which may require rapid response, investigation, or intervention, such as detection of reportable diseases.

Praedico can combine disparate technologies and has the potential to act as a go-between for VHA and other governmental agencies to facilitate collaboration and enhance assessment of other potential public health threats. It’s able to adapt to other agencies’ technologies, such as the Department of Defense [28], allowing communication across institutions and organizations.

## Results

### System attributes

Information reviewed by SMEs for 9 system attributes suggests that 3 attributes are strengths, 4 are generally good but could be improved and 2 are relative limitations of Praedico (Table 1).

**Table 1:** Summary Assessment of Surveillance Attributes for Praedico Surveillance System* in the Veterans Health Administration

Surveillance Attribute	Definition^**a**^	Qualitative Judgment
Simplicity Flexibility Stability	System design, use & ease of operation Can adapt to changing needs & technology Reliability & availability of the system	Major Strengths
Timeliness Representativeness Sensitivity Predictive Value Positive	Speed of the system, how fast data is available Degree to which the surveillance population is represented Proportion of true cases identified Proportion of reports that are true cases	Relative Strengths
Data Quality Acceptability	Data completeness & validity User’s willingness to participate in the system	Relative Limitations

^a^Adapted from Centers for Disease Control and Prevention’s “Updated Guidelines for Evaluating Public Health Surveillance System [19]

#### Simplicity

**Fig. 2:**
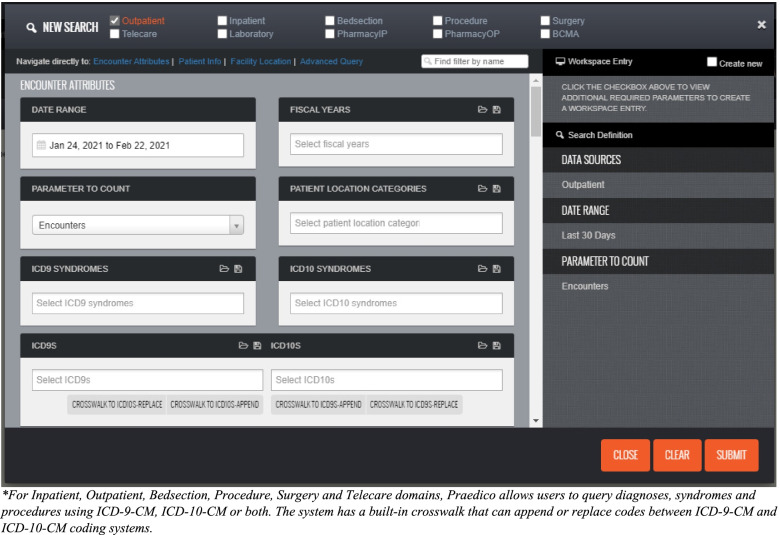
Praedico’s Query Builder*: Users select data domain(s) and criteria to include when building a query

**Fig. 3:**
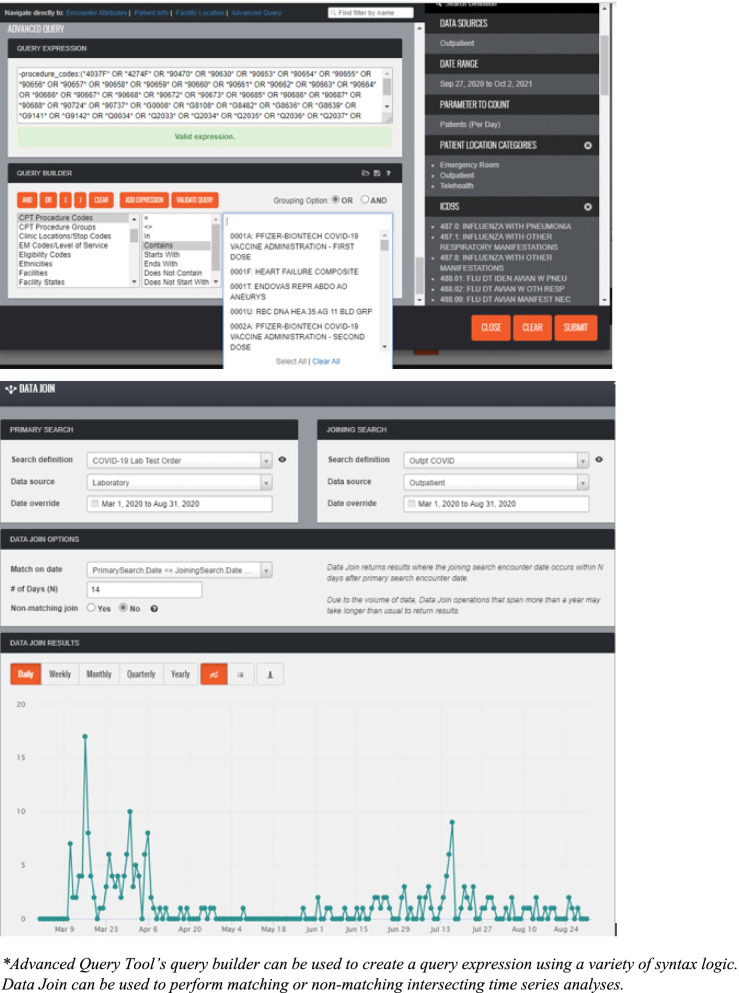
Praedico’s Advanced Query (top) and Data Join (bottom) features allow for complex queries and analyses*

Simplicity refers to structure and ease of system operation. Praedico is a complex system but with a user-friendly interface that does not require knowledge of a programming language. Instead, users select the data source or domain(s) to query and specify search criteria using dropdowns and wildcard expressions (Fig. 2). This provides time savings for routine querying and allows for less experienced users to perform queries without the need to write query expressions. Queries can be saved, refined, and executed on demand. This allows data needed on a routine basis to be retrieved quickly and consistently. An imbedded advanced query tool and data join feature allows more experienced users to create custom syntax for complicated queries and perform intersecting time series analyses (Fig. 3). This is important for complex epidemiologic questions, special studies and data requests, as well as some PHPO epidemiological investigations which need to link data from different clinical domains.

**Fig. 4:**
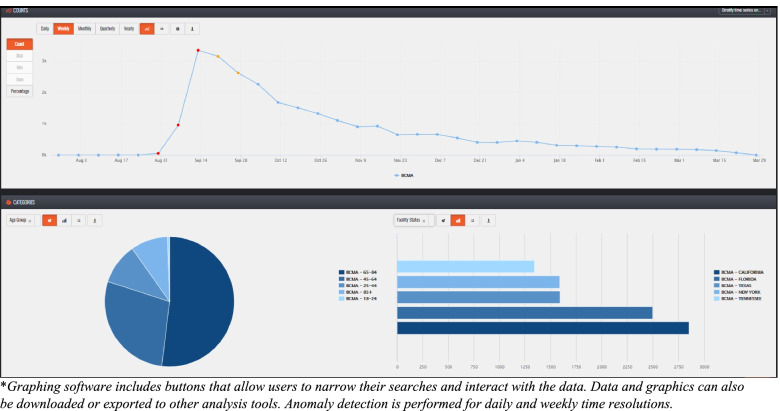
Praedico’s Graphing Tools* include times series, pie and bar graphs, tables and percentage calculations

**Fig. 5:**
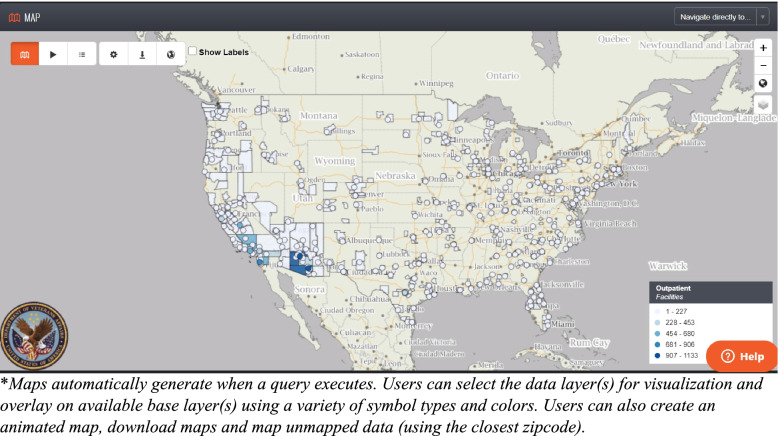
Praedico’s Geospatial Mapping Tools* enable users to visually interact with their dataset

Data analysis capabilities are built into the system, including time series, pie and bar graphs, table generation, percentage calculations, anomaly detection, data stratification, and data joins for combining multiple queries. (Fig. 4). Data can also be exported into other applications such as Excel, R, SAS®, Tableau®, Python, and others. Data visualization includes geospatial mapping (Fig. 5). Data discovery, graphing and visualization tools enables users to get a better sense of the distribution of a dataset and provides opportunities to assess trends or monitor a disease over time or geography.

#### Flexibility

Flexibility reflects the way a system adapts to changing information needs, technology, or operating conditions. Highly flexible systems adapt with relatively little time, personnel or additional funds and can be integrated with other systems. Praedico is data source agnostic and scalable. It can be adjusted to pull data from multiple sources including HL7, flat files, VistA, SQL databases and big data stacks (such as HDFS) and has the capability to harvest data from commercial EHR systems (e.g. Cerner Millennium® and Epic®), as well as unstructured sources (e.g. social media and news outlets). Data can be directly imported into the user interface and exported from any of the three system layers. Praedico has demonstrated flexibility by successfully adding new data domains from different sources over time, including the VHA’s telephone triage (DSHI TriageXpert™) system. Because Telephone triage data reside in a separate database and are not available in VistA or CDW, Praedico is the only way for PHPO epidemiologists to access this important data source. Triage data captures information on a range of illnesses and severity, occurs before patients access other health care resources or seek in-person care, and thus is important data for early event detection and situational awareness. This is particularly important for augmenting VHA’s influenza and other disease surveillance [29].

Since its launch in 2015, Praedico has been adapted to meet new PHPO influenza surveillance needs by supplying inpatient, outpatient, telephone triage, ILI and vaccine data for biweekly influenza surveillance reports and studies [29]. In 2019, Praedico began producing an automated, weekly report of influenza-related hospitalizations, intensive care admissions and deaths that eliminates much of the manual case review needed previously. During the emergence of Zika virus, Praedico was leveraged to track Zika laboratory results via a weekly data pull for on-going surveillance across VHA and to monitor hospitalizations, neurologic complications and deaths following Zika virus infection [35, 36]. More recently during the COVID-19 pandemic, Praedico data was used to develop a SARS-CoV-2 machine learning prediction model combining vital sign values, laboratory, and imaging results [39] and supplied data on COVID-19 hospitalizations and complications among Veterans with SARS-CoV-2 compared to hospitalized patients with influenza [37]. Praedico was also used for an internal evaluation of COVID-19 testing practices because the performing laboratory location (including send-out tests) could be quickly added to Praedico laboratory domain [40]. Although the system has demonstrated a good deal of flexibility, incorporation of additional data domains or major system modifications may incur additional costs. For example, current work to add additional immunization data required a contract modification and supplemental funding.

Praedico is flexible in terms of hardware and software requirements. It’s based on a scalable modular architecture that can run on commodity hardware. The Cloudera infrastructure supporting Praedico can serve a broader team’s raw data needs using Cloudera components (e.g. Pig, Hive and Apache Phoenix). These tools provide a platform for experimenting with the viability of incorporating new data domains inside Praedico’s data rubric and the ability to independently run and verify queries generated by the application.

Praedico was designed to allow various IT platforms within VHA to communicate with one another to create a central pool of data for surveillance. Praedico’s data-ingestion services seamlessly connects and consumes data from VHA’s heterogeneous databases. Praedico’s data lake repository architecture is designed to work on structured, semi-structured and unstructured data. As a result, it is ideally suited to work with and adapt to any data model, including newer standards such as Observational Medical Outcomes Partnership (OMOP) Common Data Model and Fast Healthcare Interoperability Resources (FHIR). For potential data sharing, Praedico could tap into other systems and directly integrate data sources to allow for simple exchange of data, rather than requiring agencies to adopt the same or similar systems.

#### Stability

Stability focuses on reliability and availability of the surveillance system. Overall, we found system stability very high, particularly on the application side. Praedico runs on clusters of commodity hardware for distributed processing, load balancing, high availability, and failover support. There are rare occasions where a data domain load will fail and need to be re-extracted from VistA or issues loading data from a specific VHA facility. To monitor reliability, users can navigate to the “System Status” section of Praedico to review data load status for each data domain, including daily incremental load status and any known data issues. These can include site-specific issues requiring re-extraction or reload. When issues are resolved a notation is added. For any large data extraction or availability issues, a banner will appear at the top of the application alerting users to data availability issues. A review of system reliability found that across all domains, approximately 0.01% of files fail and cannot be re-extracting in time to be available to users on the day that it should be. A review of system uptime from May 2018-February 2021 found that Praedico was available 99.5% of the time during regular business hours, with all downtimes traced back to infrastructure problems (Table 2).

**Table 2:** Praedico System Outages and Causes, Veterans Health Administration, May 2018 – February 2021

Outage	Length	Reason	Type	End User Affected
7/28/18	2 days	OIT^a^ hardware upgrade	Planned	No, non-business hours
8/18/18	2 days	OIT network update	Planned	No, non-business hours
9/11/18	2 days	Hardware repairs, storage array	Planned	Yes, during business hours
10/19/18	4 h	OIT network outage (for upgrade)	Planned	No, non-business hours
1/11/19	3 h	Hardware repairs, storage array	Planned	Yes, during business hours
3/13/19	4 h	OIT local network outage	Unplanned	Yes, during business hours
4/13/19	2 days	Host migration to OIT management	Planned	No, non-business hours
6/10/19	½ day	VA campus/city-wide power outage	Unplanned	No, non-business hours
6/27/19	4 h	Praedico moved to new OIT servers	Planned	Yes, during business hours
9/14/19	2.5 h	OIT datacenter/network maintenance	Planned	No, non-business hours

^a^*OIT* VA Office of Information & Technology

Stability is also reflected in resilience to system changes. In 2015, Praedico seamlessly adopted the ICD-10-CM and ICD-10-PCS coding systems, while maintaining ICD-9-CM system for historical records. The user interface allows users to query ICD diagnoses, procedures or syndromes using either or both coding systems. It has built-in crosswalk functionality with general equivalence mapping between ICD-9-CM and ICD-10-CM that can be used to append or replace codes from one system to the other (Fig. 2).

#### Timeliness

Timeliness considers the speed between steps in the system and how quickly data is made available. Praedico is currently configured to extract and process VHA clinical data once daily based on the current license parameters, which means that users can typically query data as recently as the day prior to the current date. System alerts for pre-defined syndromes, designated reportable diseases and user-created custom alerts also run once daily. In comparison, VHA data is sent hourly to CDC BioSense and is loaded and available in NSSP ESSENCE within 4-5 hours. Data in CDW production is updated once daily. Increasing the frequency of data pulls across all data domains in Praedico requires a higher network bandwidth than currently supported in VHA. However, the application allows pulling in of targeted domains more frequently, should users require it.

Near-real-time data upload allows users to identify actionable data in a timely manner for appropriate response or mitigation efforts. For all domains, data is available in Praedico within 1 day of the triggering event. Generally, this is when an encounter is created or updated. Because outpatient data are not available for extraction until at least one diagnosis or procedure code has been assigned there can be a minor lag in data availability. For inpatient domain, some admission records are not captured in daily incremental runs due to the way record sequence numbers are assigned. This is mitigated with a full historical extract of inpatient files each weekend, but can lead to a lag of a week or more for some inpatient records.

To quantitatively assess speed of the system, a series of 5 diagnosis and syndromic queries were compared against analogous queries in NSSP ESSENCE. To account for possible variations in network speed, the queries were run during three distinct time periods. The time to execute and download query results was measured (Table 3). Run times were faster in Praedico for all queries, regardless of the time of day performed. The time to download was similar, although slightly faster overall in NSSP ESSENCE.

**Table 3:** Query execution and download times for Praedico vs. National Syndromic Surveillance Program (NSSP) ESSENCE System^a^

Time of Day (Pacific Time)	Average Praedico Execution Time	Average ESSENCE Execution Time	Average Praedico Download Time	Average ESSENCE Download Time
9 am - 11 am	6 s	19 s	1 min, 38 s	43 s
11 am - 1 pm	7 s	18 s	29 s	46 s
1 pm – 3 pm	4 s	17 s	39 s	47 s
**Overall**	5 s	18 s	53 s	45 s

^a^Queries consisted of 5 equivalent queries during each time frame: 2 ICD-10-CM diagnosis (Influenza and COVID-19) and 3 syndrome queries (Fever, Gastrointestinal and Influenza-like-illness) from emergency department/urgent care settings and results were downloaded to Microsoft Excel

#### Representativeness

Representativeness refers to how well a system accurately describes occurrence of health events over time, and distribution in the population. VHA serves approximately 9 million enrollees out of over 14 million who are currently eligible to receive care from VHA. Enrollees are predominately older males and there is little to no pediatric data. However, enrollment of younger Veterans is increasing, and surveys have found that 62% of the over 1.9 million Veterans from Operation Enduring Freedom, Operation Iraqi Freedom and Operation New Dawn (OEF/OIF/OND) have obtained healthcare from VHA [45]. Praedico captures data for individuals who receive care within the VHA, and limited data for Veterans who received care outside the VHA system. Praedico is generally representative of the Veteran population receiving care in VHA however, VHA surveillance data may not be generalizable to the broader U.S. population. Nevertheless, for some surveillance data (such as influenza surveillance) we found that VHA data are highly correlated with national CDC surveillance data [29].

The system pulls outpatient, inpatient, bed section, pharmacy orders, Bar-Coded Medication Administration (BCMA), clinical chemistry laboratory (Lab-Chem), clinical microbiology laboratory (Lab-Micro), surgery, immunization, and procedure data as direct VistA extracts. For most domains, data are available from January 1, 2010 to present (full Lab-Chem data are available from January 2015 onward, with test types limited from January 2010-December 2014, and immunization table data from 2019 onward). Cost constraints have precluded ingestion of additional historical data for Praedico at this time. There is also a data gap in Telephone Triage data from November 2016-June 2017 due to a migration from regional to a single centralized server. Data in CDW are available back to 1999, which is an advantage when additional historical data are needed.

Only limited non-VHA care data (including DoD and community care) are available in Praedico. Although the data in Praedico encompass most critical public health surveillance domains, Praedico does not currently pull in other important clinical datasets and domains such as radiology, vital signs, problem lists, critical care management data (e.g. Picis or CareVue), pathology, transfusion, or provider progress notes. Although CDW contains some of these domains, it’s also missing data in key domains that are present in Praedico. Notably, in the fall of 2015, a safety communication was issued reporting an association of Nontuberculous Mycobacterium with heater-cooler devices (used to warm or cool blood and organs during surgeries) [46]. VHA PHPO epidemiologists collaborated with VA National Center for Patient Safety to identify potential infections among Veterans exposed to these devices. Praedico was used because Mycobacterial laboratory data are incomplete in CDW [41]. Praedico has subsequently been used for other Mycobacterial investigations and Tuberculosis reviews [42–44]. A summary of the availability of key data domains across the various platforms is presented in Table 4.

**Table 4:** Availability of key VHA data domains: Praedico, Corporate Data Warehouse (CDW), NSSP ESSENCE and VA ESSENCE

Data Domain	Praedico (2010-present)^**a**^	CDW (1999-present)	NSSP ESSENCE (August 2018-present)	VA ESSENCE (2005-2015, retired)
Emergency Dept (ED) & Urgent Care (UC) Encounters	✓ (missing chief complaint & disposition)	✓	✓	✓ (missing chief complaint & disposition)
Outpatient Encounters (other than ED/UC)	✓	✓	X	✓
Inpatient/Hospitalizations	✓	✓	X	✓
Inpatient Bed Section	✓	✓	X	✓
Patient Problem List	X	✓	✓	X
Procedure (CPT and ICD-9/10-PCS)	✓	✓	X (In progress)	✓
Surgery	✓	✓	X	✓
Laboratory (Chemistry)	✓	✓ (missing performing laboratory field)	X	X
Laboratory (Microbiology)	✓	✓ (Mycobacteria incomplete)	X	X
Pathology (Autopsy & Surgical Specimen)	X	✓	X	X
Bar-coded Medication Administration (BCMA)	✓	✓	X	X
Outpatient Pharmacy (Orders & Refills)	✓	✓	X	X
Inpatient Pharmacy (Orders)	✓	✓	X	X
Provider Notes	X	✓	X	X
Telephone Triage (*DSHI TriageXpert*™ System)	✓	X	X	✓
Immunization Table	✓ (In progress)	✓	X	X
Radiology Reports	X	✓	X	X
Vital Signs	X	✓	✓	✓
Patient Demographics (Gender, Race/ethnicity, Age, Birth/Death Date)	✓ (Race/ethnicity not available for some data domains)	✓	✓ (Death Date not available)	✓ (Race/ethnicity not available for some domains)

*CDW* Corporate Data Warehouse, *NSSP* National Syndromic Surveillance Program, *CPT* Current Procedural Terminology, *ICD-9/10-PCS* International Classification of Disease, 9th and 10th Revisions-Procedure Coding System

^a^limited laboratory chemistry data from 2010 to 2014 and telephone triage data from 11/2016-6/2017

#### Data quality

Data quality measures the completeness and validity of the data in a system. A strength of Praedico is that data are extracted directly from the VistA source. There are multiple extractors with failover and redundancy capabilities and monitoring to check that extractors are operating correctly. Praedico’s extract, transform, load (ETL) module performs the suite of data quality checks needed for ensuring data fidelity to its source. For completeness, each extracted file has a header and trailer that are checked during processing. If these are missing, the file is deleted and re-extracted. At each stage of ETL, the input versus output record count must match, otherwise an alert is generated. There is also manual sampling and a statistical trend analysis to monitor the expected range of records and deviations are investigated. For validity, the ETL pipeline verifies that all expected fields are present in the correct order and that the data type for each field is correct. Additional checks include the extraction engine keeping track of the total records extracted from VistA in each extract file which is further tallied in the ETL pipeline during processing. Assessments of Praedico data quality, including completeness and validity were performed via a record-level comparison to CDW and raw VistA data extracts by data domain in 2019 (Table 5) and re-assessed in 2020.

**Table 5:** Data Quality Comparison of Praedico and VA’s Corporate Data Warehouse (CDW), 2019

Data Domain	CDW Records	Praedico Missing Records (%)
Bar Code Medication Administration (BCMA)	136,831,255	108 (0.00008%)
Outpatient Encounters	127,046,092	9274 (0.0073%)
Inpatient (Hospitalizations)^a^	705,011	141 (0.02%)
Laboratory (Chemistry)	559,244,279	4,311,773 (0.771%)
Laboratory (Microbiology)	1,798,704	0 (%)
Pharmacy (Outpatient)	6,386,096	16,668 (0.261%)
Pharmacy (Outpatient-Refills)	262,023,921	98,285,173 (37.51%)

^a^The inpatient domain comparison included data from 2010 to 2018. All other domains are 2018 data only

Data quality was in general high. Regarding data completeness, the review identified missing records in the Lab-Chem domain (~ 1% missing records) and Pharmacy Outpatient-Refills (~ 38% missing records, which required a reload of historical data from 2010 to 2019 for all facilities). Users also reported data completeness issues with missing test name field in some Lab-Micro records prior to 2013 and there is incomplete data from the Spokane VA Medical Center which was the first VHA facility to migrate from VistA/CPRS to Cerner EHR for which Praedico data access has not been established. The 2020 re-assessment found improvement in most domains, except for BCMA where approximately 3.3% of CDW records were missing in Praedico. Some deficiencies were also identified with data validity where the content of Praedico records did not match CDW. These discrepancies are generally related to records that are altered, updated, or deleted after initial transmission. Praedico identifies modified records using an incremental lookback window. The window varies by domain and must be balanced with time needed for daily extraction of new records. Praedico has expanded its lookback window for most domains (previously 5000 records per domain) and added a longer window to run every weekend (Table 6). This improved but did not completely resolve identified data completeness and validity issues. CDW, on the other hand, has access to VistA journal, an audit trail of all changes made to VistA files, which Praedico does not currently have permission to read. Data quality issues are discussed further in the following two sections.

**Table 6:** Praedico’s Daily and Weekend Lookback Settings, by Data Domain

Data Domain	Daily incremental lookback	Weekend incremental lookback
Bar Code Medication Administration (BCMA)	Last 25,000 records	Last 300,000 records
Outpatient Encounters	Last 15,000 records	Last 75,000 records
Inpatient (Hospitalizations)	Last 5000 records	Last 50,000 records
Laboratory (Chemistry)^a^	Last 14 days	Last 185 days
Laboratory (Microbiology)	Last 63 days	Last 185 days
Pharmacy (Outpatient)	Last 5000 records	Last 5000 records
Pharmacy (Outpatient-Refills)	Last 7 days	Last 7 days
Pharmacy (Inpatient)	Last 14 days	Last 38 days

^a^Daily incremental extract examines last 60 days of records (185 for weekend incremental extract) based on specimen collection date, then within this window, selects any new or changed records within the last 14 days (either collection date or report completion date)

#### Sensitivity

Sensitivity is best described as the proportion of cases of a disease that the system identifies as well as the ability of the system to detect outbreaks or monitor changes in cases over time. To perform an evaluation of sensitivity, the ability of Praedico to correctly identify influenza hospitalizations was assessed. Influenza diagnosis codes (ICD-10-CM: J09-J11) were queried in Praedico for the 2017-18 and again for the 2019-20 influenza seasons and compared to equivalent queries developed for CDW. EHR reviews were performed to determine why cases were missed. Praedico correctly identified 10,424 of the 10,426 hospitalizations found in CDW during 2017-18 and 7018 of 7079 hospitalizations during 2019-20 (sensitivity = 0.999 and 0.991, respectively). Two hospitalizations missed in 2017-18 were modified records where the influenza diagnosis code was not initially present when the record was extracted by Praedico. Seven missing records from 2019-20 were patients hospitalized outside the VHA system. The remaining missing records in 2019-20 were determined to have an “out of order” record identifier in the VistA file which prevented Praedico’s VistA extract from identifying them.

An analysis to evaluate the sensitivity of Praedico data for the Lab-Chem domain was performed for January 2018 - March 2019. Laboratory results are frequently utilized to identify disease cases or to confirm a diagnosis. During this time there were over 750 million Lab-Chem records, with a total of 265,972 (0.04%) missing records in Praedico. A more detailed analysis of the test result value field was performed for 14 different VHA facilities (4 facilities during January - December 2016, 5 facilities during January - December 2017, and 5 facilities during January 2018 - February 2019). The number of incorrect test results in Praedico ranged from 3 to 669 for the 1.6 million to 12 million tests performed for the facilities and time periods analyzed. Sensitivity was > 0.999 for all analyses performed. Again, incorrect test values occurred because records were subsequently modified but not updated in Praedico. This issue and approaches to minimize its impact were described in the Data Quality section.

For outbreak detection, a comparison of Praedico with the retired VA ESSENCE was performed. A validation set combining 17 million DoD and 25 million VHA records was used for the initial evaluation [28] and a second analysis used 513 million VHA patient records was performed [47]. For the influenza-like-illness (ILI) syndrome, both applications displayed the same number of ILI cases. However, for the time period June 2014 – May 2015, only 62% of Praedico syndromic alerts directly correlated with VA ESSENCE, suggesting that Praedico identified some ILI outbreaks/clusters that were not detected by ESSENCE and that the alerts in Praedico were not simply a subset of VA ESSENCE alerts. Praedico also demonstrated higher seasonal sensitivity (with increased ILI alerting in December and January and more likely representing actual influenza activity), adjusting for seasonality using historical and seasonal information, whereas VA ESSENCE alerts were more uniformly distributed throughout the year [47]. Sensitivity of the Praedico system is additionally enhanced through the access to *DSHI TriageXpert*™ telephone triage data which captures mild illnesses among individuals that may never seek in-person care and thus represents illness data that is not available via CDW or other active VHA surveillance systems.

#### Predictive value positive (PVP)

PVP describes the proportion of disease cases in a system that are true cases. Here, we again evaluated the ability of Praedico to correctly identify influenza-coded hospitalizations in VHA as well as false positives in the laboratory domains. As described above, influenza hospitalizations were queried during two influenza seasons and compared to results from CDW. A total of 4 out of 10,428 hospitalizations in 2017-18 and 1 out of 7079 in 2019-20 identified in Praedico were not present in CDW (PVP > 0.999 for both seasons). Record review against raw VistA files determined these represented false positives because the influenza code was deleted from the record during a subsequent modification, but not updated in Praedico.

The Lab-Chem analysis for the 14 VHA facilities described above found false positives ranging from 32 to 25,450 for the 1.6 million to 12 million lab tests performed per site. PVP was > 0.98 for all analyses performed. For the Lab-Micro domain during January 1, 2010 – April 10, 2019 we identified 21,714 false positives out of 20,923,588 tests performed (PVP = 0.9989). In these domains, false positives were records deleted from VistA but not from Praedico. A further broad review of all records in 7 major Praedico data domains found that false positives ranged from a low of 0.1% (BCMA, Lab-Chem, Lab-Micro, Outpatient Pharmacy and Outpatient Pharmacy- Refills) to a high of 1% (Inpatient).

False positive alerts are a problem that plague many surveillance systems. The ability of the BDaP data extraction component of Praedico to scrub data following a set of business intelligence rules minimizes the number of extraneous alerts. False alerting results in lost time and resources and creates the impression among users that most alerts are irrelevant. To assess alerting, Praedico was compared to VA ESSENCE and found to be somewhat more accurate with fewer false positive alerts [47]. This translated to less alerting fatigue for users and lessened the need for frequent and intensive manual review of surveillance data.

#### Acceptability

Acceptability refers to the willingness of persons and organizations to participate in the surveillance system. Veterans do not need to enroll or opt-in to having their clinical data made available to Praedico [48]. Because the data are used for public health operational activities, VHA Office of Research Oversight considers analysis of these data as operational and not research in VHA [49]. Features of Praedico that contribute to acceptability among users of the system are simplicity, speed, flexibility, and other key attributes described earlier, such as alerting, automation, analytic and visualization capabilities, and a straightforward user interface. One additional element of Praedico that increases acceptability is the user workspace. Here users can run and save queries that span multiple datasets, set alerts, request and share reports, and more. Saved queries can be executed, modified, duplicated, deleted, or shared. Workspaces are customizable, creating a dynamic dashboard that organizes queries in user-defined groupings.

In general, however, acceptability is a limitation as Praedico is only utilized by a small number of PHPO epidemiologists. However, the system has relevant applications for a variety of health system personnel including epidemiologists, informaticians, clinicians, researchers, infection preventionists, antimicrobial stewards and other data analysts. Spin-offs of Praedico using similar technology have been developed by Bitscopic Inc. and some are currently in use in VHA including PraediGene (laboratory workflow, financial and DNA analysis tool), PraedAlert (clinical surveillance and antimicrobial stewardship tool), PraediCare (analytic and reporting system for high acuity clinical areas) and PraediTrial (clinical trial recruitment/enrollment tool). Praedico is a major expenditure for VHA’s PHPO group and only available when VHA has an active license for its use. Costs must be budgeted carefully as any major modification requires contract modification and supplemental funding. Also, separate IT funding must be secured for system maintenance and infrastructure needs. Ultimately, cost may be the primary factor working against acceptability, especially in a climate of restricted program budgets. Examples of other factors and limitations users have reported which affect acceptability are fewer years of historical data and limited number of data domains available, constraints on the number of records that can be downloaded and exported, missing tests names for Lab-Micro data prior to 2013, missing test types for Lab-Chem data prior to 2015, missing Emergency and Urgent Care chief complaint data, and not being able to filter numeric values for laboratory test results. Furthermore, adapting existing CDW queries to run in Praedico may not be straightforward or possible, which leads some users to continue using CDW in situations where they already have a working query.

## Discussion

Too often, public health surveillance systems in use today look at data in silos analyzing just one or a few datasets at a time. Data from multiple sources are difficult to combine which limits the ability to find relevant correlations and evaluate trends for meaningful surveillance. To facilitate inter-agency cooperation, systems need to be able to adapt to and integrate disparate data sources and technologies and handle big data. Such a system must be flexible, simple to use, reliable, timely, accurate, and intelligent.

This evaluation found that overall Praedico is relatively easy to use, with rapid query generation and runtimes and enables analysis across single or multiple data sets. Praedico can be adapted to pull data from many source types in near real-time. Such queries are less costly to scale and maintain due to the Cloudera ecosystem (including a HDFS). Praedico stores data efficiently and uses commodity hardware with a scale-out model rather than a traditional enterprise architecture with a scale-up model. Data querying in Praedico requires no specific programming expertise because the system uses a query builder interface. Lastly, data can be easily graphed, mapped, analyzed and shared with key decision makers.

Praedico has several limitations. Although the system is generally representative it is missing relevant clinical data domains which are either in progress or have not been specifically funded for inclusion. There are only a limited number of users and some notable acceptability constraints. Also, there is limited data available on care received outside of VHA, including DoD. DoD clinical data may become more readily available to applications like Praedico with the VA and DoD Cerner EHR modernization and alignment in the coming years [50]. Praedico contains less historical data compared with CDW which impacts representativeness. Although Praedico harnesses big data technologies it has not been able to fully take advantage of some of its inherent capabilities due to VA IT infrastructure constraints. Moving Praedico to a cloud environment could improve some of the current infrastructure challenges. Finally, although data quality is high, several issues were identified including the need to correct modified and deleted records through expansion of the lookback window and adoption of a journaling approach. The evaluation methods also had some limitations. It should be noted that quantitative attributes of Praedico were compared to data from VA ESSENCE, CDC NSSP ESSENCE and CDW as a “gold standard”. CDW is a data warehouse and not truly an end-user application such as Praedico, VA ESSENCE or CDC NSSP ESSENCE. This evaluation did not attempt to assess Praedico against non-VA systems (other than VHA data within CDC NSSP and limited DoD comparisons). For future evaluations, including sensitivity and PVP assessments, it may be valuable to compare Praedico’s performance to non-VA systems, including DoD systems and to assess additional aspects of the system that were not included or fully considered in this analysis.

The CDC 2001 public health surveillance systems evaluation guidelines [19] remain a valuable framework for assessing surveillance systems. Now that 20 years have passed, it would be beneficial to have additionally updated and expanded guidelines. First, a more comprehensive list of attributes with greater flexibility, prioritization, and guidance as to how to select the best complement of attributes for review would be helpful. This would allow the review process to better align with objectives of the evaluation and to consider nuances or constraints of a system as well as stakeholder-specific system needs that impact which attributes are most relevant to evaluate and optimize [23]. For example, an assessment of value (including IT and system costs, funding, and impact) would have been useful for this evaluation. Additionally, effectiveness including an assessment of how well the system can identify meaningful correlations between different data sets or data domains, would be another important area for evaluation in our system. These along with organizational assessments (such as data management and security) and additional functional assessments (such as inter-agency data sharing), we would recommend for inclusion in future evaluations of Praedico.

## Conclusion

System reviews performed by experienced users can provide objective and important feedback on implementation and use of surveillance data and can help provide focus for future improvements in areas which need to be strengthened. Praedico is an effective public health surveillance application that leverages leading technology advancements in software architecture and big data to create a system designed to gather and process large amounts of data. In addition to routine surveillance activities, it’s designed to be an early warning system, facilitate data gathering for public health investigations and provide situational awareness. Praedico enables VHA public health decision makers to react to a health crisis in a timely manner and share information with key stakeholders, while accessing data reliably and securely.

## Data Availability

The data that support the findings of this study are available from the corresponding author upon reasonable request.
